# A Genome-Wide Association Study to Identify Novel Candidate Genes Related to Low-Nitrogen Tolerance in Cucumber (*Cucumis sativus* L.)

**DOI:** 10.3390/genes14030662

**Published:** 2023-03-06

**Authors:** Bowen Li, Aimin Wei, Xueqiang Tong, Yike Han, Nan Liu, Zhengwu Chen, Hongyu Yang, Huaxiang Wu, Mingjie Lv, Ning Ning Wang, Shengli Du

**Affiliations:** 1 College of Life Science, Nankai University, Tianjin 300071, China; 2 Cucumber Research Institute, Tianjin Academy of Agricultural Sciences, Tianjin 300192, China; 3 State Key Laboratory of Vegetable Biobreeding, Tianjin 300192, China; 4 Institute of Germplasm Resources and Biotechnology, Tianjin Academy of Agricultural Sciences, Tianjin 300061, China; 5 College of Agricultural Science, Nankai University, Tianjin 300071, China

**Keywords:** cucumber, genome-wide association study (GWAS), nitrogen limitation

## Abstract

Cucumber is one of the most important vegetables, and nitrogen is essential for the growth and fruit production of cucumbers. It is crucial to develop cultivars with nitrogen limitation tolerance or high nitrogen efficiency for green and efficient development in cucumber industry. To reveal the genetic basis of cucumber response to nitrogen starvation, a genome-wide association study (GWAS) was conducted on a collection of a genetically diverse population of cucumber (*Cucumis sativus* L.) comprising 88 inbred and DH accessions including the North China type, the Eurasian type, the Japanese and South China type mixed subtype, and the South China type subtype. Phenotypic evaluation of six traits under control (14 mM) and treatment (3.5 mM) N conditions depicted the presence of broad natural variation in the studied population. The GWAS results showed that there were significant differences in the population for nitrogen limitation treatment. Nine significant loci were identified corresponding to six LD blocks, three of which overlapped. Sixteen genes were selected by GO annotation associated with nitrogen. Five low-nitrogen stress tolerance genes were finally identified by gene haplotype analysis: CsaV3_3G003630 (CsNRPD1), CsaV3_3G002970 (CsNRT1.1), CsaV3_4G030260 (CsSnRK2.5), CsaV3_4G026940, and CsaV3_3G011820 (CsNPF5.2). Taken together, the experimental data and identification of candidate genes presented in this study offer valuable insights and serve as a useful reference for the genetic enhancement of nitrogen limitation tolerance in cucumbers.

## 1. Introduction

Cucumber is one of the most widely cultivated vegetables worldwide. According to data from Faostat, the global production of cucumbers in 2020 was approximately 90 million tons, of which China produced approximately 80%. Whether fresh or frozen, China’s cucumbers and gherkins represent significant export commodities in terms of export volume and foreign exchange earnings. Notably, the export value of these commodities reached an impressive 63.321 million USD in 2019. This underscores the critical importance of these vegetables to the agricultural sector and the economy of China. As a high-yield vegetable variety, the application of nitrogen fertilizer is very important in the cultivation process of cucumber. Nitrate, which is the primary source of nitrogen fertilizer for cucumbers, not only has an effect on the growth [1] and the amount of fruit production [2], but it also plays an essential role in the resistance of cucumbers to disease [3]. Since it is difficult for nitrate to remain in the soil for an extended period of time, regular topdressing is required, which not only increases planting costs but also has negative environmental effects such as eutrophication of water bodies [4,5]. Improving cucumber tolerance to low-nitrogen stress and boosting nitrogen use efficiency are effective solutions to this issue [6]. However, low-nitrogen stress tolerance and nitrogen efficiency are governed by numerous metabolic, developmental, and environmental signaling networks throughout the plant life cycle. To increase nitrogen efficiency and improve tolerance to low-nitrogen stress, nitrogen uptake, transport, and assimilation, as well as a series of signaling networks should be considered [7].

Increasing tolerance to low-nitrogen stress and enhancing nitrogen usage efficiency have been extensively studied in rice and other essential food crops, and many important QTLs and key functional genes have been unearthed. By evaluating the absorption efficiency of nitrate and ammonium in indica and japonica rice, Chu’s team uncovered the high-nitrate utilization gene NRT1.1b [8], and discovered that NRT1.1b boosts nitrogen efficiency via rice rhizosphere microflora [9]. Using genome-wide association analysis, Wan’s group revealed a nitrate transporter OsNPF6.1^HapB^ that promotes rice nitrate uptake and increases rice yield under low-nitrogen conditions [10]. OsTCP19 works as a regulatory factor to block the expression of the tiller-promoting gene DLT, thereby realizing the regulation of rice tiller development [11]. Through a comparative investigation of the transcriptome and metabolome of maize and rice leaf tissue, Zhou’s team identified the gene OsDREB1C, which increases photosynthesis and nitrogen efficiency; OsDREB1C overexpression lines displayed a phenotype of increased grain production as a result of improved photosynthetic capacity and more effective carbon and nitrogen transfer from source to sink [12]. However, few investigations have been conducted on cucumber’s low-nitrogen tolerance and nitrogen-efficient genes. Qiu et al. [1] discovered that CsIVP adversely regulates high-nitrate-affinity transporters NRT2.1 and NRT2.5 and redistribution transporters NRT1.7, NRT1.9, and NRT1.12 in the presence of low-nitrogen stress. The transgenic strain CsIVP-RNAi is resistant to low-nitrogen stress [13]. Gao et al. discovered that cucumber nitrate transporter CsNRT2.1 knockout lines had a decreased number and length of lateral roots, as well as impaired constitutive and inducible high-affinity nitrate transport systems at low nitrate concentrations [14]. Exploring genes for low-nitrogen stress tolerance and nitrogen efficiency will greatly help to create new germplasms and cultivars with high nitrogen efficiency.

GWAS is an efficient technique for identifying genes related to trait variation [15]. By integrating genotype and phenotype, statistical analysis is performed at the population level, and the genetic variation markers that are most likely to influence the characteristic are screened out on the basis of statistics or significant *p*-values [16]. With the development of GWAS technology, GWAS analysis models have been further developed and optimized, such as EMMA, GEMMA, and other models that are optimized to improve the calculation speed, MLMM and SUPER models that are optimized to improve the calculation accuracy, and the FarmCPU and BLINK models that increase accuracy on the basis of computing speed [17]. In recent years, GWAS has been utilized extensively to investigate genes related with complex traits in rice [13], cotton [18], corn [19], and other plant species. Currently, there is no research on mining cucumber genes for low-nitrogen stress resistance or nitrogen efficiency using GWAS. We conducted GWAS using the resequencing data of 88 inbred cucumber materials and the phenotypes of cucumbers grown under low-nitrogen and normal-nitrogen circumstances to identify candidate genes for low-nitrogen stress resistance in cucumber.

## 2. Materials and Methods

### 2.1. Plant Materials and Treatment

A total of 88 DH and inbred cucumber lines from Cucumber Research Institute of Tianjin Academy of Agricultural Sciences (P R China) were used as genetic material. The lines primarily consisted of North China type, South China type, Eurasia type, and Japanese type cucumbers. The seeds were soaked at 55 °C for 15 min, and then at room temperature for 3 h, before being placed in a germination incubator at 28 °C for 12 to 18 h to germinate. Germinated seeds were sown in the 50-hole seedling tray with peat substrate and cultured in greenhouse conditions in February of 2020. After the first genuine leaf fully unfurled, the plants were transplanted into vermiculite-filled flowerpots. The size of the flowerpot was 19 cm × 18 cm (diameter and height), the void density of vermiculite was 2.5 g/cm^3^, and one seedling was transplanted to each flowerpot. The plants were irrigated with Yamazaki cucumber culture fluid and low-nitrogen culture fluid based on the Yamazaki cucumber culture formula. For the nutrient solution, see Table S1. Three plants were grown in triplicate for treatment.

### 2.2. Phenotype Measurement and Parameter Calculation

Phenotypic data including plant height, dry weight, and total N content were measured 25 days after nutrient solution treatment. The dry weight of the aboveground part was measured by cutting the aboveground plant before subjecting it to a high temperature treatment at 103 °C in an oven for 30 Min, and then further drying in the oven at 80 °C to a constant weight. The dried plant aboveground parts were fully ground, and the total amount of nitrogen was assayed through the Kjeldahl method by the Institute of Agricultural Resources and Environment of Tianjin Academy of Agricultural Sciences [20]. The nitrogen accumulation level (NA), nitrogen uptake efficiency (NUpE), and nitrogen utilization efficiency (NUtE) of the plant were calculated through the dry weight and nitrogen content of the plant and the nitrogen used in the irrigation nutrient solution, as shown in the following formulas [21,22]:

NA=Total N content of plant×Dry weight of plant,

NUpE=Total N content of plant ÷N supplied,

NUtE=Dry weight of plant ÷Total N content of plant.

The GGally package ^21^ was used to analyze and visualize the correlation of phenotypic data. The normality analysis of the selected phenotypes was performed using SPSS (v26.0.) and the normality was mainly determined by the Shapiro–Wilk test, the Kolmogorov–Smirnov test, and the Z-score of kurtosis and skewness. FactoMineR (v2.7) were used for principal component analysis of phenotypes [23].

### 2.3. SNP Genotype Data Acquisition

The genotype data of 88 cucumber accessions with an average sequencing depth of 30.673× were completed by Beijing Novogene Corporation Inc. through the Illumina system. There were 4,218,416 SNPs in the genotype data of 88 materials. SNPs were called using GATK software [24], after alignment to the *Cucumis sativus* L. cv. 9930 reference genome [25]. SNP data were filtered by VCFtools (v0.1.16), allowing max-missing 0.9, maf 0.05, minDP 2, maxDP 1000, minQ 30, minGQ 0, min-alleles 2, and max-alleles 2. After filtering, there were a total of 583,718 SNPs on the seven chromosomes for downstream analyses. The filtered data were annotated and analyzed using Snpeff [26].

### 2.4. Population Genetic Evolution

Iqtree2 was used to construct a phylogenetic tree using the maximum likelihood method for the filtered SNP files [27]. The base substitution model was the GTR model, and the step size was set to 1000. Plink [28] was used for principal component analysis and group structure analysis on a population. The number of principle components was set to 10, with the first principal component’s explanation ratio being 56.2% and the second principal component’s explanation ratio being 9.48%. Two to ten components were set for population structure analysis, and the ideal component was chosen on the basis of the cross-validation error. Population genetic evolution methods were performed following protocols [29].

### 2.5. GWAS

The genome-wide association study was implemented with model BLINK [30] in GAPIT package [31]. The above ground part dry weight ratio (DWR), plant height ratio (HR), NA ratio (NAR), NupE ratio (NupER), NutE ratio (NutER), which were the ratios of their values under low nitrogen to the values under normal-nitrogen conditions, respectively, and the nitrogen content under low-nitrogen conditions (LNC) were used as the phenotypic traits for GWAS. Due to the complexity of the phenotypes, the significance threshold was set to 1 × 10^−5^ to avoid false negatives caused by high thresholds.

### 2.6. Candidate Gene Selection

LDBlockShow was used to determine the LDBLOCK associated with significant SNPs and the genes in the interval [32]. The candidate genes were annotated using eggNOG-mapper [33] while referring to the function of the homologous genes of the candidate genes in *Arabidopsis*. The genes involved in nitrate or amino-acid metabolism, assimilation, and absorption were chosen. Candihap [34] was used to perform haplotype analysis on candidate genes, and genes with differences in different haplotypes were selected. Gene structure mapping was performed using IBS 1.0.2 [35].

## 3. Result

### 3.1. SNPs Characteristics in Cucumber Genome and Cucumber Population Distribution

After the sequencing results of 88 inbred cucumbers were called, genome files with 583,718 SNPs were obtained, and the results were annotated with snpEff, which showed that the average rate of the total SNPs was 361; the rate of chromosome 5 was the highest, and the rate of chromosome 3 was the lowest (Table S2, Figure S1). Furthermore, 31.569% of SNPs were located in the intergenic region, followed by the upstream and downstream regions of the gene, accounting for 28.957% and 25.962%, respectively. The exon region contained 2.141% of the SNPs, the SNPs that caused missense mutations accounted for 43.684% of the total number of SNPs in the exon region, and the SNPs that caused changes in the start codon and stop codon accounted for 1.031%.

**Figure 1:**
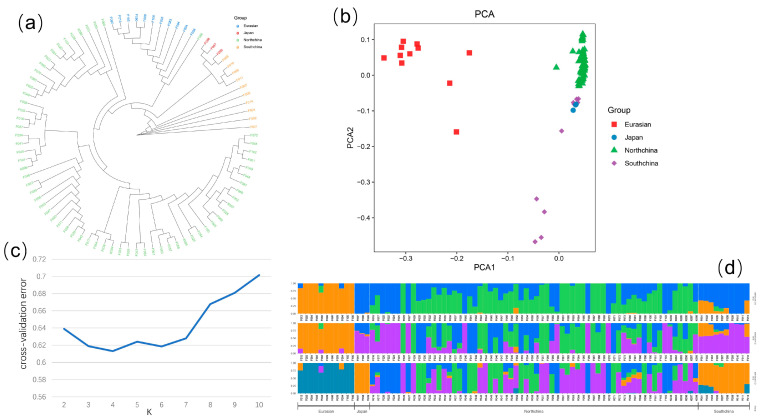
Genetic diversity and population structure of the studied cucumber accessions. (**a**) Phylogenetic trees constructed based on maximum likelihood method for 88 lines. (**b**) PCA of 88 cucumber lines, where the *y*-axis quantifies subgroup membership, and the *x*-axis shows the different accessions. (**c**) Cross-validation error value of K = 2–10 in population structure analysis. (**d**) Population structure composition of 88 cucumber lines at K = 3–5.

Using iqtree to construct a phylogenetic tree, it was determined that the population of cucumbers was classified into four subtypes: the North China type, the Eurasian type, the Japanese and South China type mixed subtype, and the South China type subtype (Figure 1a). The results of the PCA analysis were consistent with those of the phylogenetic tree (Figure 1b). Population structure analysis revealed that four population clusters (K = 4) represented the optimal model, although the difference with K3 was modest.

### 3.2. Evaluation of Cucumber Population Tolerance to Low Nitrogen

**Figure 2:**
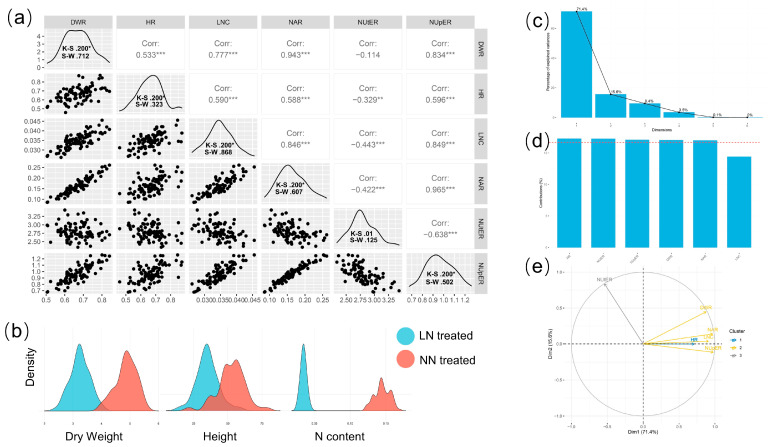
Phenotypic analysis of 88 cucumber lines. (**a**) Phenotypic correlation and normality analysis of cucumber lines, where the number in the upper triangle is the Pearson correlation coefficient; * *p* ≤ 0.05, **, *** = Significant Pearson correlation coefficient at 0.01 and 0.001 probability levels. (**b**) The density distribution of dry weight, plant height, and nitrogen content of 88 cucumber lines under low-nitrogen and normal-nitrogen nutrient conditions. (**c**) Principal component explanation rate of dimensions, where the first four are coordinates for the variables, correlations between variables and dimensions, Cos2 for the variables, and contributions of the variables. (**d**) Contribution of variables to dimensions 1-3. (**e**) PCA of variables.

The aboveground part dry weight, plant height, and nitrogen content of plants under different nutrient solution treatments were measured, and the nitrate assimilation ratio (NAR), the nitrogen uptake efficiency ratio (NupER), and the nitrogen use efficiency ratio (NutER) of all treatments were calculated (Table 1). There existed significant variations in nitrogen content, dry weight, and plant height between the low-nitrogen treatment and normal-nitrogen control (Figure S3 and Figure 2b). The nitrogen content of plants under low-nitrogen treatment (LNC), DWR, HR, NAR, NUpER, and NUtER were used to evaluate the low-nitrogen tolerance level of 88 cucumber lines. The results indicated that the phenotypic variation coefficients of 88 cucumber lines ranged from 8.048% to 23.940%, with NAR having the highest value. As shown in Figure 2a, the low-nitrogen stress tolerance traits of all 88 lines conformed to the normal distribution verified by Kolmogorov–Smirnov (K–S) test and Shapiro–Wilk (S–W) test; although the K–S value of NUtER was less than 0.05, the Z-scores of skewness and kurtosis were 1.89 and 0.49, which conformed to normal distribution. The scatter distribution of the phenotypes is represented by the lower triangle area of Figure 2a, whereas the upper triangle region indicates the correlation. The data indicated that all the traits except for NUtER exhibited positive correlations of varied degrees. The correlation values were comparable to those of phenotypic PCA (Figure 2e). Principal component analysis was performed using coordinates for the variables, correlations between variables and dimensions, and Cos2 for the variables as the principal components. The variables NAR, NupER, NAR, LNC, and DWR were the most prominent main components. The contribution of NUtER and DWR was greater in the second major component. HR was the primary variable in the third principal component. The contribution of HR, NUtER, NupER, NAR, and DWR was relatively substantial when combined with the three primary components (Figure 2d). In addition, eight low-nitrogen sensitive lines (LNS) and 11 low-nitrogen tolerant lines (LNT) were screened on the basis of the phenotypic data.

### 3.3. GWAS

**Figure 3:**
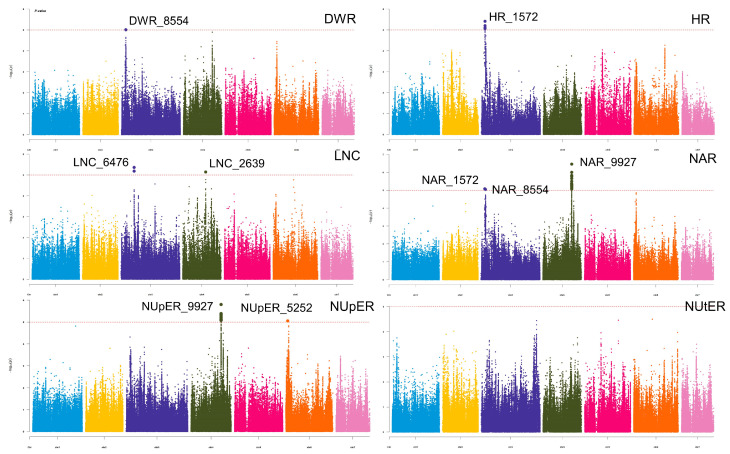
GWAS Manhattan plots of low-nitrogen tolerance in six traits. the color blue, yellow, dark blue, black, rose red, orange, and pink represent each SNP located in chromosomes 1–7, respectively. The vertical axis represents the calculated *p*-value of each SNP, which is −log10. The horizontal axis represents the chromosome where the SNP is located.

GWAS was performed using SNP data and six traits (Figure 3). The filtered SNPs with allele frequencies less than 0.05 were used for GWAS analysis in the BLINK model. The BLINK model identified nine significant SNPs associated with traits, respectively. Significant SNPs were found on chromosomes 3, 4, and 6, with chromosome 3 having the highest number, followed by chromosome 4 and finally chromosome 6. These significant loci were classified as six LDBLOCKs (Table 2), with DWR_8554 from the DWR trait and NAR_8554 from the NAR trait co-locating to a repeating LD block, HR_1572 and NAR_1572 co-locating to the same LD block, NupER_9227 and NAR_9227 classified as the same LD block.

### 3.4. Analysis of Candidate Genes by GO Annotation

A total of 61 candidate genes involved in 6 LDBLOCKs (File S1) were functionally annotated using eggNOG-MAPPER (File S1). Furthermore, 32 genes were annotated by GO, and a total of 16 genes involved in nitrate transport, metabolism, and synthesis were screened. The detailed information was shown in Table 3.

### 3.5. Analysis of Candidate Genes in LD blocks

We first selected the LD blocks which were repeatedly localized: BLOCK 8554, block 1572, and block 9227. Block 8554 was colocalized by DWR_8554 and NAR_8554, block 1572 was colocalized by HR_1572 and NAR_1572, and block 9927 was colocalized by NAR_9227 and NupER_9927. Genes from each block were analyzed according to the distribution of LNT and LNS in different haplotypes combined with functional annotation.

#### 3.5.1. Candidate Gene Analysis in LD block 8554

**Figure 4:**
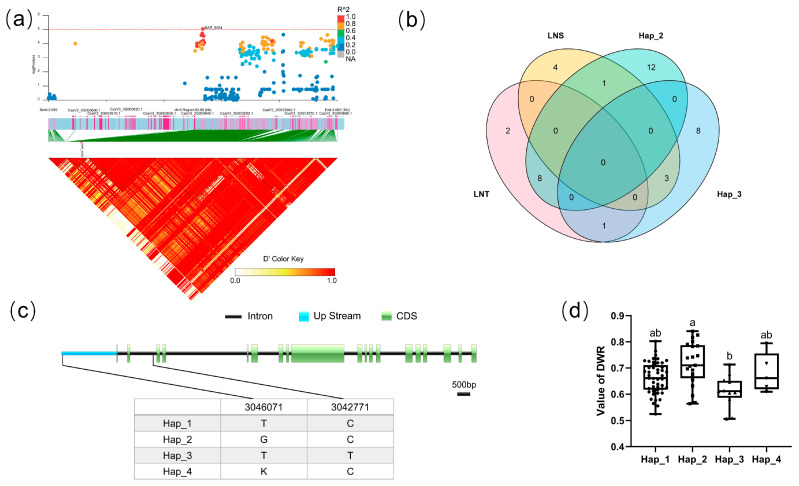
Identification of the low-nitrate tolerance gene at block 8554. (**a**) Manhattan map (top) and LD heatmap (bottom) of the block 8554. (**b**) Hap_3 and Hap_2 of CsaV3_3G003630 in LNS and LNR. (**c**) SNP variation of the candidate gene CsaV3_3G003630 among four haplotypes. (**d**) Haplotype difference of CsaV3_3G003630, different letters meant there was significant difference among groups (*p* < 0.05).

For block 8554, the candidate region (Chr.3: 3,046,071–3,048,993 bp) contained three candidate genes (F4a), including CsaV3_3G003630, CsaV3_3G003640, and CsaV3_3G003650. Haplotype analysis revealed that the two SNPs of CsaV3_3G003630 were located in the upstream promoter region and intron region of the gene, respectively. There mainly existed four haplotypes (Figure 4c). Analysis of the LNT and LNS contained in different haplotypes of CsaV3_3G003630 revealed that LNT:LNS was 8:1 in Hap_2 and 1:3 in Hap_3 (F4b). There was a significant difference between Hap_2 and Hap_3 in DWR, whereby Hap_2 tended to be more tolerant to low-nitrogen stress, while haplotype Hap_3 was more sensitive to low-nitrogen stress (Figure 4d). CsaV3_3G003630 was described as a DNA-directed RNA polymerase subunit. The homologous gene of CsaV3_3G003630 in *Arabidopsis* is AtNRPD1, which plays a role in plant immunity through DNA methylation [36]. DNA methylation plays a crucial role in response to environmental stimuli [37,38,39]. Whether CsNRPD1 plays a role in cucumber low-nitrogen stress tolerance through DNA methylation needs further research.

#### 3.5.2. Candidate Gene Analysis in LD Block 1572

**Figure 5:**
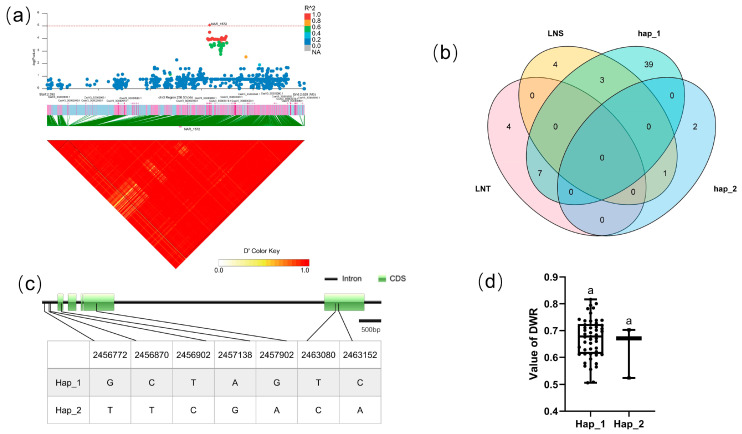
Identification of the low-nitrate tolerance gene at block 1572. (**a**) Manhattan map (top) and LD heatmap (bottom) of the block 1572. (**b**) Raf and Alt of CsaV3_3G002970 in LNS and LNR. (**c**) SNP variation of the candidate gene CsaV3_3G002970 among 2 haplotypes. (**d**) Haplotype difference of CsaV3_3G002970, different letters meant there was significant difference among groups (*p* < 0.05).

For BLOCK 1572, the candidate region (Chr.3: 2,413,014–2,613,011 bp) contained 13 candidate genes (Figure 5a). CsaV3_3G002970 was annotated as an *Arabidopsis* NFP6.3/NRT1.1 homologous protein. As the first discovered nitrate transporter and nitrate sensor in plants, NPF6.3/NRT1.1 can detect varying levels of exogenous nitrate signals [40], participate in the primary nitrate response (PNR), and activate plant hormone synthesis and signaling of cytokinins and auxins, allowing for rapid nitrate uptake and assimilation [41,42]. There are seven SNPs in the CsaV3_3G002970 gene, four of which are located in the CDS, all of which are synonymous mutations. Cucumber populations are mainly divided into two haplotypes, Among the 88 cucumber populations (Figure 5c), 49 lines are hap_1, and three lines are hap_2. Analysis of the LNT and LNS contained in different haplotypes of CsaV3_3G002970 revealed that the LNT:LNS ratio is 7:3 in Hap_1 and 0:1 in Hap_2 (Figure 5b), although there is no significant difference in the DWR value between Hap_1 and Hap_2 (Figure 5d).

#### 3.5.3. Candidate Gene Analysis in LD Block 9927

**Figure 6:**
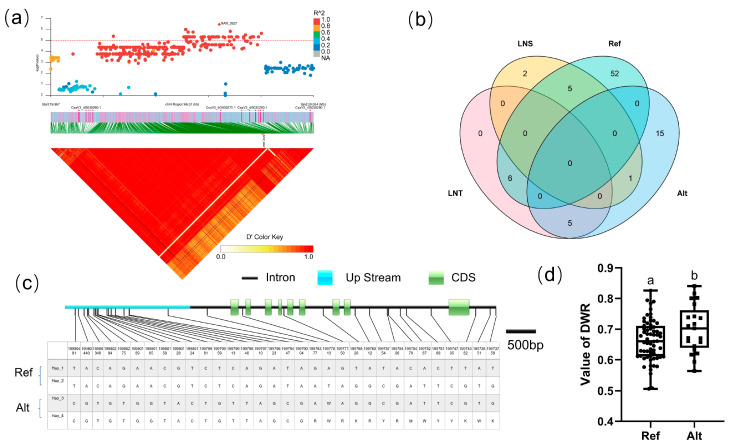
Identification of the low-nitrate tolerance gene at block 9927. (**a**) Manhattan map (top) and LD heatmap (bottom) of the block 9927. (**b**) Raf and Alt of CsaV3_4G030260 in LNS and LNR. (**c**) SNP variation of the candidate gene CsaV3_4G030260 among two haplotypes. (**d**) Haplotype difference of CsaV3_4G030260, Different letters meant there was significant difference among groups (*p* < 0.05).

For block 9927, the candidate region (Chr.4: 19,970,542–20,024,272 bp) contained five candidate genes (Figure 6a): CsaV3 4G030250, CsaV3 4G030260, CsaV3 4G030270, CsaV3 4G030280, and CsaV3 4G030290. There existed four haplotypes (Hap_1–4) of CsaV3_4G030260; the promoter region of Hap_1–2 is the same as that of the reference genome, while the promoter region of Hap_3–4 had a completely different SNP distribution in the reference genome (Figure 6c). In order to explore the influence of the promoter region on the gene, Hap_1–2 were classified as the Ref group, and Hap_3–4 were classified as the Alt group. Analysis of the LNT and LNS contained in different groups of CsaV3_4G030260 revealed that the LNT:LNS ratio was 5:1 in the Alt group. The DWR value of the two groups was significantly different, which means that the change in the promoter region of CsaV3_4G030260 may increase the tolerance of cucumber populations to low-nitrogen stress. CsaV3_4G030260 is a homologous gene of *Arabidopsis* AtSnRK2.5. SnRK is a kind of Ser/Thr protein kinase with high conservation. According to sequence similarity, structure, and function, it can be divided into three subfamilies: SnRK1, SnRK2, and SnRK3, among which SNRK2 is involved in osmotic stress and the abscisic acid response, and plays an important role in stress signal transduction [43]. Elena Baena-González et al. found that ABA promotes the interaction between SnRK1 and TOR protein, triggering the inhibition of TOR activity. Further experiments proved that PP2C mediates the protein interaction between SnRK1 and SnRK2, while ABA inhibits the SnRK2–PP2C–SnRK1 protein interaction. These results suggest that SnRK2, an important regulator of the ABA signaling pathway, may indirectly regulate TOR function by regulating the activity of SnRK1 [44]. Yu’s group found that CsSnRK2.5 in Camellia was strongly induced by ABA [45], and the role of SnRK2.5 in salt stress tolerance was reported successively [46,47].

#### 3.5.4. Candidate Gene Analysis in LD Block 2639

**Figure 7:**
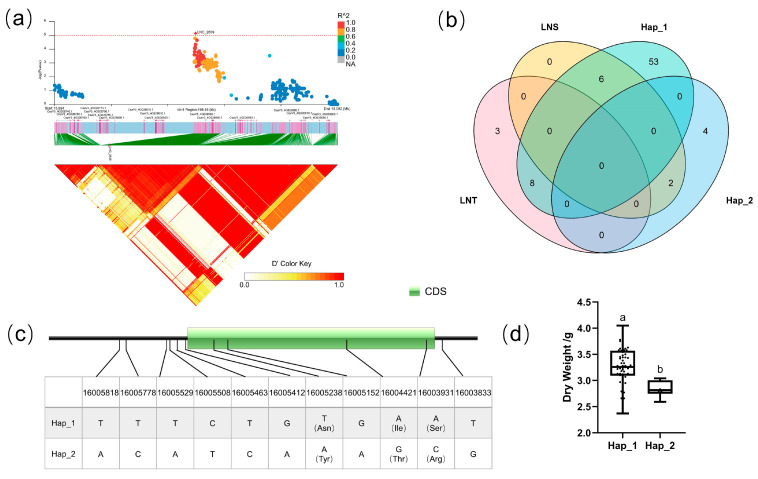
Identification of the low-nitrate tolerance gene at block 2639. (**a**) Manhattan map (top) and LD heatmap (bottom) of the block 2639. (**b**) Raf and Alt of CsaV3_4G026940 in LNS and LNR. (**c**) SNP variation of the candidate gene CsaV3_4G026940 among 2 haplotypes. (**d**) Haplotype difference of CsaV3_4G026940, Different letters meant there was significant difference among groups (*p* < 0.05).

For block 2639, the candidate region (Chr.4: 15,910,369–16,009,194 bp) contained six candidate genes (Figure 6a): CsaV3_4G026750, CsaV3_4G026760, CsaV3_4G026920, CsaV3_4G026930, CsaV3_4G026940, and CsaV3_4G026950. There were four SNPs in the CDS region of CsaV3_4G026940, and the three SNPs caused missense mutations. The dry weight of two types of haplotype in CsaV3_4G026940 showed a significant difference under low-nitrogen conditions, which reflected that the Hap_2 haplotype of this gene was more sensitive to low-nitrogen stress (Figure 7d). CsaV3_4G026940 was annotated as the protein PLANT CADMIUM RESISTANCE like. Homologous genes of CsaV3_4G026940 in *Arabidopsis* include PLAC8 family proteins such as AT3G18470. Through a comparison of gene expression differences between the *Arabidopsis* nitrogen limitation adaptation mutant (*nla*) and wildtype, Zhu et al. found that the expression of AT3G18470 only changes in NLA nitrogen deficiency but not in wildtype [48].

#### 3.5.5. Candidate Gene Analysis in LD Block 6476

**Figure 8:**
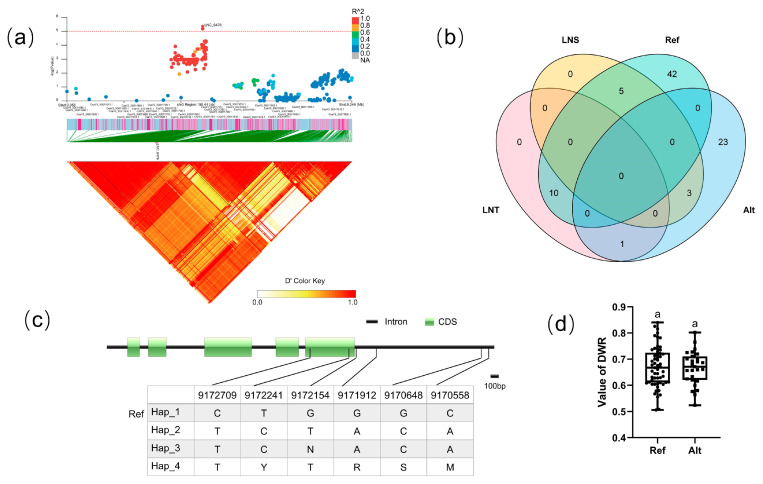
Identification of the low-nitrate tolerance gene at block 2639. (**a**) Manhattan map (top) and LD heatmap (bottom) of the block 2639. (**b**) Raf and Alt of CsaV3_3G011820 in LNS and LNR. (**c**) SNP variation of the candidate gene CsaV3_3G011820 among 4 haplotypes. (**d**) Haplotype difference of CsaV3_3G011820, Different letters meant there was significant difference among groups (*p* < 0.05).

For block 6476, the candidate region (Chr.3: 9,132,044–9,168,717 bp) contained nine candidate genes (Figure 8a). There existed four haplotypes (Hap_1–4) of CsaV3_3G011820 (Figure 8c). To explore the effects of differences from the reference genome, Hap_1 was classified as the Ref group, which was the same as the reference genome, while the Alt group totally differed from the reference genome. Analysis of the LNT and LNS contained in different groups of CsaV3_3G011820 revealed that the LNT:LNS ratio was 10:5 in the Ref population, but 1:3 in the Alt population (Figure 8b), although there was no significant difference in DWR values between the two groups (Figure 8d). The *Arabidopsis* homolog of this gene is peptide transporter AtNPF5.2, which is involved in responses to wounding, virulent bacterial pathogens, and high NaCl concentrations [49].

## 4. Discussion

Nitrogen is a critical component of several vital plant components, including chlorophyll, amino acids, and nucleic acids, making it one of the most crucial elements for plant growth and development [50,51,52]. Nitrate nitrogen is lost from the soil due to plant absorption, irrigation or rainwater [53], resulting in low nitrogen use efficiency. Enhancing plant resistance to low-nitrogen stress and nitrogen use can minimize planting expenses and nitrate pollution. For the genetic basis of cucumber tolerance to low-nitrogen stress, we treated seedling cucumber populations with low nitrogen. Eleven LNT and 8 LNS were screened by phenotypic data evaluation; these extreme materials can provide genetic materials for subsequent omics research. Sixteen candidate genes were annotated relative to nitrate by GO annotation, and five genes were further selected. CsaV3_3G003630 (CsNRPD1), CsaV3_3G002970 (CsNRT1.1), CsaV3_4G030260 (CsSnRK2.5), CsaV3_4G026940, and CsaV3_3G011820 (CsNPF5.2) were identified through haplotype analysis, according to the ratio of LNS and LNR in the cucumber population.

To investigate the genetic basis of cucumber’s low-nitrogen stress tolerance, we subjected cucumber seedlings to low-nitrogen and normal-nitrogen circumstances. The height, dry weight, and nitrogen content were measured, and the corresponding values of NA, NUpE, and NUtE were calculated. Subsequently, to accurately reflect the phenotypic response to low-nitrogen stress, the ratio of low nitrogen to normal nitrogen was selected as the trait for GWAS. The nitrogen concentration of the low-nitrogen nutrient solution used in the experiment was formulated to be 3.5 mM, whereas that of the normal-nitrogen nutrient solution was 14 mM, thus providing the basis for NUpE calculations. However, it is imperative to note that the actual nitrogen supply levels were slightly below these values. Additionally, this highlights why the ratio of nitrogen content between low nitrogen and normal nitrogen was not considered as the trait for GWAS analysis, as it is similar to NUpER.

The gene CsaV3_3G002970 (CsNPF 6.3) has garnered extensive attention in the field of biology due to its potential impact on nitrogen efficiency. In this study, we performed a haplotype analysis to examine the potential influence of this gene on phenotypic traits. Unfortunately, our findings did not reveal any significant differences in these traits. This lack of significant results may be due to the limited sample size, which resulted in the absence of certain haplotype varieties in our statistical analysis. The differences in the distribution of the main haplotypes Hap_1 and Hap_2 between LNT and LNS indicate to a certain extent that expanding the material population may help reveal the influence of SNPs at different positions on CsNPF 6.3. CsaV3_4G030250 (CsSnRK2.5), a member of the SnRK2 family, plays a critical role in plant stress response. In this study, we performed a haplotype analysis of the four main haplotypes of CsaV3_4G030250. Our results showed no significant differences among these haplotypes. However, on the basis of the promoter region analysis, we found that Hap_1 and Hap_2 were similar and were classified as the “Ref” group, due to their consistency with the reference genome sequence. Conversely, Hap_3 and Hap_4 showed consistency in their promoter regions and contained SNPs that were different from the reference genome sequence; they were, thus, classified as the “Alt” group. A haplotype analysis of these two groups revealed that the DWR value of the Alt group was significantly higher than the Ref group, suggesting that changes in the promoter region SNP may have altered the regulation of CsSnRK2.5 by an unknown transcription factor. Further studies are required to fully understand the underlying mechanisms of this regulation. CsaV3_4G026940 including three missense mutations in its coding region and the resulting haplotypes exhibited significant differences in DWR value. These differences may be attributed to the substitution of Ile to Thr at position 327. A SMART domain analysis revealed that this amino acid is located in the transmembrane domain. Further investigation is required to determine whether this substitution is indeed the underlying cause of the observed differences in DWR value. CsaV3_3G011820 (CsNPF5.2), a key player within the peptide transporter family, plays a crucial role in mediating plant responses to stress. In this study, we conducted a haplotype analysis of this gene and found that there were no significant differences among its haplotypes. However, based on consistency with the reference genome, we divided the haplotypes into two groups: “Ref” and “Alt”. The Ref group comprised 57 lines and was represented by Hap_1, while the Alt group contained six haplotype varieties among 27 lines. Our results revealed differences in the distribution of LNT and LNS between these two groups. The insignificant difference in haplotype analysis may have been due to insufficient sample size.

The regulation in response to low-nitrogen stress is a very complex regulatory network involving not only the absorption, transport, and assimilation of nitrate, as well as the regulatory network associated with plant abiotic stress. The low-nitrogen stress tolerance network can be studied using a multi-omics approach. In addition to genome-wide association studies (GWAS), RNA-Seq, a typical omics research tool, is widely employed in the investigation of low-nitrogen stress tolerance [1,54,55]. On the basis of genome-wide analysis and the combination of the transcriptome, metabolome, and proteome, it is feasible to examine the low-nitrogen stress network in greater depth [56,57].

## 5. Conclusions

In this study, we identified nine loci in six LD blocks that are associated with low-nitrogen stress tolerance in cucumber through GWAS analysis. We also annotated 16 candidate genes using GO analysis, and further explored five putative cucumber genes related to low-nitrogen stress through haplotype analysis. Among these candidate genes, the homologs of CsaV3_3G002970 (CsNRT1.1) and CsaV3_3G011820 (CsNPF5.2) in *Arabidopsis* or rice have been previously studied as members of the nitrate transporter family. Additionally, the homolog of CsaV3_4G026940 in *Arabidopsis* is known to be involved in the response to low-nitrogen stress, while CsaV3_3G003630 (CsNRPD1) and CsaV3_4G030260 (CsSnRK2.5) are involved in plant response to abiotic stress. However, the specific role of these genes in cucumber low-nitrogen stress tolerance requires further experimental verification.

Our results were obtained using GWAS analysis of 88 cucumbers, which provided valuable insights into the genetic basis of cucumber low-nitrogen stress tolerance. However, due to the complexity of the nitrogen metabolism network, further research combining other omics methods may identify additional genes related to low-nitrogen stress. Additionally, expanding the number of species used for GWAS analysis may help optimize these results. Our findings have significant application value for developing new cucumber varieties with tolerance to low-nitrogen stress and high yield potential.

## Figures and Tables

**Table 1:** Phenotypic variation of 88 cucumber lines.

Trait	Min	Max	Mean	SD	CV (%)
DWR	0.510	0.840	0.6697	0.073	10.936
HR	0.460	0.870	0.6658	0.081	12.206
LNC	0.030	0.050	0.0347	0.004	11.297
NAR	0.090	0.260	0.1637	0.039	23.940
NUtER	0.680	1.260	0.9655	0.136	14.111
NUpER	2.340	3.440	2.7947	0.225	8.048

**Table 2:** Details of Lead SNPs and LD BLOCK.

Lead SNP Name	LD Block	Pos	Chr	PVE	Maf	LD Block Range	SNPs Number	Genes Number
DWR_8554	8554	3,048,554	chr3	9.84 × 10^−6^	0.31	3,046,071–3,048,993	27	3
HR_1572	1572	2,541,572	chr3	3.88 × 10^−6^	0.28	2,413,014–2,613,011	870	13
LNC_6476	6476	9,146,476	chr3	4.34 × 10^−6^	0.26	9,132,044–9,168,717	123	9
LNC_2639	2639	15,992,639	chr4	7.19 × 10^−6^	0.16	15,910,369–16,009,194	176	6
NAR_1572	1572	2,541,572	chr3	8.40 × 10^−6^	0.28	2,413,014–2,613,011	870	13
NAR_8554	8554	3,048,554	chr3	9.06 × 10^−6^	0.31	3,046,071–3,048,993	27	3
NAR_9927	9927	20,009,927	chr4	3.49 × 10^−7^	0.22	19,970,542–20,024,272	462	5
NUpER_9927	9927	20,009,927	chr4	1.58 × 10^−6^	0.22	19,970,542–20,024,272	462	5
NUpER_5252	5252	1,255,252	chr6	8.91 × 10^−6^	0.39	1,060,742–1,260,725	621	25

The table above shows the nine lead SNPs of LDBLOCK, as well as their positions, chromosomes, Maf, PVE, the block range, and the number of SNPs and genes included.

**Table 3:** Candidate genes by GO annotation.

Gene ID	LD Block	Description	GO Annotation
CsaV3_3G002970	1572	Nitrate transporter	GO:0010167, GO:0015112, GO:0015706, GO:0071705, GO:1901698
CsaV3_3G002990	1572	Adenine phosphoribosyltransferase	GO:0006807, GO:0009308, GO:0034641, GO:0044271, GO:1901564, GO:1901566
CsaV3_3G003050	1572	NAC domain-containing protein	GO:0051171
CsaV3_3G003630	8554	DNA-dependent RNA polymerase catalyzes the transcription of DNA into RNA using the four ribonucleoside triphosphates as substrates	GO:0006807, GO:0034641, GO:1901698, GO:1901699
CsaV3_3G011740	6476	U-box domain-containing protein	GO:0006807, GO:0034641, GO:1901564
CsaV3_3G011750	6476	Prp19/Pso4-like	GO:0006807, GO:0034641, GO:1901564
CsaV3_3G011820	6476	Protein NRT1 PTR FAMILY 5.2-like	GO:0010243, GO:0042886, GO:0042887, GO:0071705, GO:1901698
CsaV3_4G026760	2639	-	GO:0051171, GO:0051173
CsaV3_4G026950	2639	Regulatory-associated protein of TOR (RAPTOR1)	GO:0010243, GO:0051171, GO:0051173, GO:0071417, GO:1901698, GO:1901699
CsaV3_4G030260	9927	Belongs to the protein kinase superfamily (SNRK2.1)	GO:0006807, GO:1901564
CsaV3_6G001670	5252	WUSCHEL-related homeobox (WOX5)	GO:0051171
CsaV3_6G001680	5252	component of the eukaryotic translation initiation factor 3 (eIF-3) complex, which is involved in protein synthesis of a specialized repertoire of mRNAs and, together with other initiation factors, and stimulates binding of mRNA and methionyl-tRNAi to the 40S ribosome. The eIF-3 complex specifically targets and initiates translation of a subset of mRNAs involved in cell proliferation	GO:0006807, GO:0034641, GO:0044271, GO:1901564, GO:1901566
CsaV3_6G001760	5252	Involved in the post-translational conjugation of arginine to the N-terminal aspartate or glutamate of a protein. This arginylation is required for degradation of the protein via the ubiquitin pathway	GO:0006807, GO:1901564, GO:1901565
CsaV3_6G001800	5252	Anthranilate synthase (ASA1)	GO:0006807, GO:0009308, GO:0009309, GO:0034641, GO:0044106, GO:0044271, GO:1901564, GO:1901566
CsaV3_6G001850	5252	NF-X1-type zinc finger protein	GO:0006807, GO:0034641, GO:0044271, GO:0051171, GO:0051172
CsaV3_6G001860	5252	Dual specificity tyrosine-phosphorylation-regulated kinase	GO:0006807, GO:1901564

## Data Availability

The raw Illumina sequence reads have been deposited into the National Genomics Data Center (https://bigd.big.ac.cn/) under accession number CRA004282.

## Supplementary Materials

The following are available online at https://www.mdpi.com/article/10.3390/genes14030662/s1: Figure S1. The number of SNPs within 1 Mb window size; Figure S2. Principal component analysis of phenotypic data; Figure S3. Dry weight, height, and N content of cucumbers under different treatment; **** *p* < 0.0001; Table S1. Solution formula; Table S2. SNP distribution in cucumber chromosome. File S1: Genes in all blocks.
