# Supplementary material for: A Genome-Wide Association Study to Identify Novel Candidate Genes Related to Low-Nitrogen Tolerance in Cucumber (Cucumis sativus L.)
Source: Genes (Basel). 2023 Mar 6;14(3):662. doi: 10.3390/genes14030662 (PMC10048605; doi:10.3390/genes14030662)
Supplement: Supplementary file 1 [file genes-14-00662-s001.zip › genes-2252754-supplement Figures and Tables.pdf]

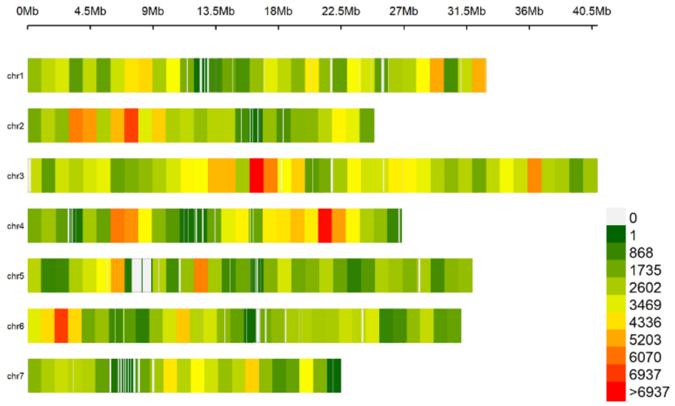

Figure S1. The number of SNPs within 1Mb Window size

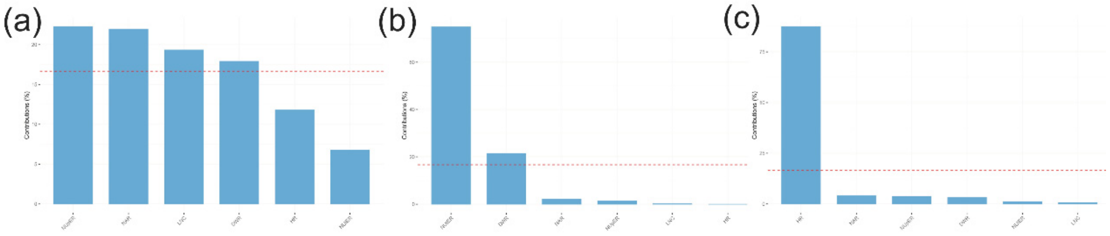

Figure S2. Principal component analysis of phenotypic data
(a) -(b) significant phenotypes in Dim1-3,

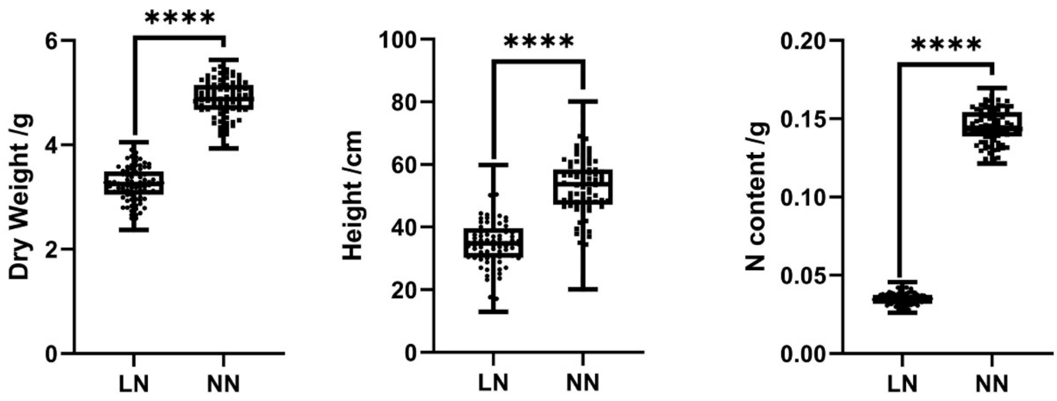

Figure S3. Dry Weight, Height and N content of cucumbers under different treatment. **** indicate significance at $p < 0.0001$

Table S1.Solution formula

Nitrogen Content	Solution A	Solution B
Low Nitrogen (3.5mmol/L N)	$Ca(NO_3)_2 \cdot 4H_2O$:413<br>$CaCl_2$:194. 25	K_2SO_4:522<br>$MgSO_4 \cdot 7H_2O$:492<br>$NH_4H_2PO_4$:115
Normal Nitrogen (14mmol/L N)	KNO_3:606<br>$Ca(NO_3)_2 \cdot 4H_2O$:826	$MgSO_4 \cdot 7H_2O$:492<br>$NH_4H_2PO_4$:115

Micronutrients should also be added in the concentrations shown below, EDTA·Fe16mg/L, H_3PO_3 3mg/L, $MnSO_4$ 2mg/L, $ZnSO_4$ 0.22mg/L, $CuSO_4$ 0.08mg/L, $(NH_4)6Mo_7O_{24} \cdot 4H_2O$ 0.5mg/L

Table S2. SNP distribution in cucumber chromosom

Chromosome	Length	Variants	Variants rate
1	32,926,272	89,772	366
2	24,837,039	75,231	330
3	40,877,379	133,583	306
4	26,827,763	81,194	330
5	31,913,682	68,612	465
6	31,125,843	80,689	385
7	22,466,726	54,637	411
Total	210,974,704	583,718	361
